# ‘Falling heads’: investigating reflexive responses to head–neck perturbations

**DOI:** 10.1186/s12938-022-00994-9

**Published:** 2022-04-16

**Authors:** Isabell Wochner, Lennart V. Nölle, Oleksandr V. Martynenko, Syn Schmitt

**Affiliations:** Institute for Modelling and Simulation of Biomechanical Systems, Stuttgart Center for Simulation Science, University of Stuttgart, Stuttgart, Germany

**Keywords:** Reflex behaviour, Head–neck perturbations, Motor control, Musculoskeletal model, 3D finite element modelling, Muscle modelling

## Abstract

**Background:**

Reflexive responses to head–neck perturbations affect the injury risk in many different situations ranging from sports-related impact to car accident scenarios. Although several experiments have been conducted to investigate these head–neck responses to various perturbations, it is still unclear why and how individuals react differently and what the implications of these different responses across subjects on the potential injuries might be. Therefore, we see a need for both experimental data and biophysically valid computational Human Body Models with bio-inspired muscle control strategies to understand individual reflex responses better.

**Methods:**

To address this issue, we conducted perturbation experiments of the head–neck complex and used this data to examine control strategies in a simulation model. In the experiments, which we call ’falling heads’ experiments, volunteers were placed in a supine and a prone position on a table with an additional trapdoor supporting the head. This trapdoor was suddenly released, leading to a free-fall movement of the head until reflexive responses of muscles stopped the downwards movement.

**Results:**

We analysed the kinematic, neuronal and dynamic responses for all individuals and show their differences for separate age and sex groups. We show that these results can be used to validate two simple reflex controllers which are able to predict human biophysical movement and modulate the response necessary to represent a large variability of participants.

**Conclusions:**

We present characteristic parameters such as joint stiffness, peak accelerations and latency times. Based on this data, we show that there is a large difference in the individual reflexive responses between participants. Furthermore, we show that the perturbation direction (supine vs. prone) significantly influences the measured kinematic quantities. Finally, ’falling heads’ experiments data are provided open-source to be used as a benchmark test to compare different muscle control strategies and to validate existing active Human Body Models directly.

**Supplementary Information:**

The online version contains supplementary material available at 10.1186/s12938-022-00994-9.

## Background

Head and neck injuries, such as traumatic brain injuries, concussions and whiplash-associated disorders, can occur in a multitude of scenarios varying from traffic accidents and physical assaults to sports and recreation-related collisions. The main point they have in common is that they are induced by biomechanical forces such as contact or inertial forces that are transmitted to the brain, head or upper body. The resulting injuries are widely recognized as a significant public health concern [3, 61]. Hence, it is critical to identify individual risk factors for such injuries to understand the causes and develop injury prevention strategies.

Previous studies investigating head–neck responses to perturbations conducted experiments with different methodological setups, including load dropping, release and direct impacts to the head–neck complex [29, 42, 47, 56, 67, 72]. One of these methods, the release experiment, was proposed by Ito et al. [29, 30]. They introduced this new technique for studying responses in neck muscles by exposing the head to a sudden onset of a free fall under its own weight. Using this method, they compared normal and labyrinthine-defective subjects in the supine position (extension of the head) and demonstrated that reflex responses contribute significantly to head-righting. Investigations of another research group by Portero et al. [54–56] examined the response to a similar release experiment, including preloads in both flexion and extension positions. They focused on assessing the musculotendinous stiffness of the head–neck segment but only in the first $30\,\hbox {ms}$ after the acceleration peak to avoid altered kinematics due to reflexive contributions. For a general overview of experimental studies with regard to head–neck perturbations, we refer to the systematic review of Le Flao et al. [42]. As a conclusion, they requested future studies to include neck muscle latency [${\hbox {ms}}$], neck stiffness [${\hbox {Nm/rad}}$], linear accelerations [${\hbox {g}}$] and rotational head accelerations [${\hbox {rad}/\hbox {s}^2}$] due to their potential use in assessing concussion risks.

These concussion risks are related to linear and rotational head accelerations as prevailing injury theories provided in literature [19, 60, 81] suggest. However, the magnitude of force needed to cause these injuries cannot be studied in ethically justifiable experiments. Computer simulations using musculoskeletal models provide an alternative assessment tool, additionally used in this study.

These simulations allow us to estimate the forces and moments within the body, while varying muscle activations and control strategies. To ensure that the predicted response during simulation studies using biomechanical models is biophysically valid, both correct muscle modelling, as well as bio-inspired control strategies, are crucial. Several studies [26, 57, 66] state that the muscles’ reaction alters the head kinematics and therefore, the influence of cervical muscles and their control strategy on the head–neck response can be significant.

In this contribution, we want to study this influence by presenting the results of ’falling heads’ experiments in a supine and prone position to investigate the individual responses to head–neck perturbations. Additionally, we mimicked this experimental setup in numerical simulations. Based on these setups, we quantify the kinematic, dynamic and neuronal response to head–neck perturbations and pose the question of how human diversity (such as biological sex and age) affects these quantities. Furthermore, we use the numerical model to answer the question whether and how the biomechanical response is affected by changes of the neuronal state (e.g. sensitivity to the stretching of the muscle).

The purpose of this study is to give insights in understanding individual head–neck responses to perturbations and to provide a comprehensive data set as open-source^1^ which can be directly used as a benchmark setup to compare and validate different models and controllers. The novelty of our work is twofold: first, we use a similar ’falling heads’ setup as proposed by Ito et al. [29, 30] but with two different force directions (flexion and extension) and for a larger number of healthy participants with different ages and sexes. Second, we build up a numerical model with the same setup and compare potential muscle reflex controller strategies to experimental findings.

## Results

### Kinematic, neuronal and dynamic characteristics of the reflex response

**Fig. 1:**
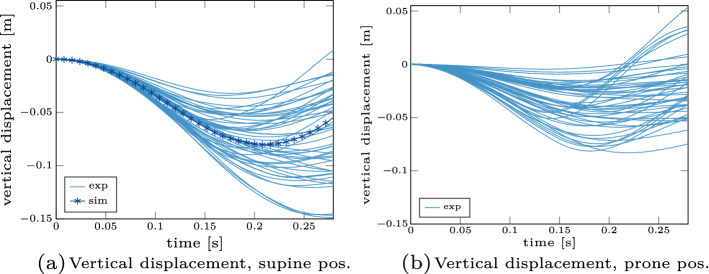
Vertical displacement of ’falling heads’ experiment. The vertical displacement of the supine (**a**) and prone (**b**) position is shown for all participants and all trials (light blue solid lines). For the supine case, we additionally show the simulation trajectory (as dark blue line with asterisks)

In this section, we present the main results in a condensed form. First, vertical displacement curves of all participants extracted from the video data are shown in Fig. 1. Additionally, the simulation curve for the supine case is given in Fig. 1a as a comparison value. Three things can be noted from the presented results: first, the range of the maximal falling height varies between participants (in the range between 3.2–$14.9\,\hbox {cm}$ for the supine case). Second, the participants tend to fall less in the prone case (range between 0.5–$8.3\,\hbox {cm}$, Fig. 1b) compared to the supine case. This difference is significant ($p < 0.01$), for a detailed overview of the statistical analysis we refer to Additional file 1: Table E3 the supplementary material E. Third, the simulated supine experiment shows a good agreement with the experiments with regard to the displacement and can predict similar head-fall kinematics in terms of both the maximum displacement as well as the general slope and timing.

The difference between peak displacements in the supine and the prone cases shows a similar trend for the peak accelerations and time to peak accelerations. An overview of all mean and standard deviations for the peak accelerations is given in Table 1. Both, the linear peak acceleration and the time to linear peak acceleration are higher in the supine compared to the prone case ($p < 0.01$). These values are comparable to literature values of similar experiments, e.g. Ito et al. [29, 30] who reported linear mean peak acceleration values of 0.76–$1.2\,\hbox {g}$ for the supine case (for the healthy participants). The same tendency of greater peak acceleration in the supine case can also be seen for the rotational accelerations ($p < 0.01$). The same increase between peak accelerations during forced flexion (prone case) and forced extension (supine case) is also reported in the literature [47, 72]. They report smaller absolute values (in the range of 12.8–$36.2\,\hbox {rad}/\hbox {s}^2$); however, they also had smaller flexion and extension angles due to a different experimental setup.

These kinematic characteristics are partly influenced by the latency time of the muscles contributing to reflexive behaviour in response to the perturbation. The mean and standard deviations for the detected EMG onset for both muscles (*SCM* and *trapezius*) are given in Table 2. The range of latency times in this study was 17.67–$86.67\,\hbox {ms}$ which is comparable to previously reported values of 18.6–$88.0\,\hbox {ms}$ for quick-release or load-dropping studies [7, 14, 29, 30, 47, 63, 72]. Furthermore, it can be noted that the *SCM* is activated faster than the *trapezius* in both cases which is also reported in Corna et al. [7], Ito et al. [30].

**Fig. 2:**
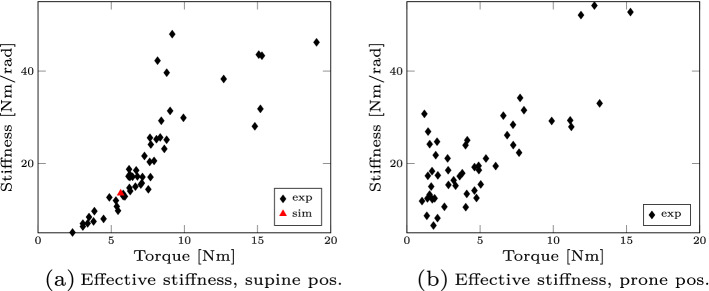
Effective stiffness. The effective stiffness of the supine position (**a**) and the prone position (**b**) is shown for all participants and all trials (black diamonds). For the supine case, we additionally show the simulation value (red triangle)

The effective stiffness represents the combination of these kinematic and neuronal reflex responses. We show this stiffness plotted over the change of torque in Fig. 2. The absolute values are comparable to previous studies which reported $22\,\hbox {Nm/rad}$ [67] or a range between 22.6–$41.3\,\hbox {Nm/rad}$ [72]. Furthermore, we see an increase of the effective stiffness for an increase in torque as also supported by Portero et al. [55, 56].

**Table 1:** Peak accelerations (given as mean ± standard deviation)

	Supine	Prone
Peak lin. acc.	− 0.7 ± 0.1 g	− 0.5 ± 0.2 g
Time to peak lin. acc.	44.0 ± 3.5 ms	36.4 ± 2.5 ms
Peak rot. acc.	62.4 ± 11.5 rad/s^2^	44.0 ± 18.2 rad/s^2^
Time to peak rot. acc.	52.1 ± 10.1 ms	57.8 ± 12.1 ms

**Table 2:** EMG latency times (given as mean ± standard deviation)

	Supine	Prone
*SCM*	$33.4 \pm 17.6\,\hbox {ms}$	$31.5 \pm 12.1\,\hbox {ms}$
*Trapezius*	$49.0 \pm 19.7\,\hbox {ms}$	$43.8 \pm 13.5\,\hbox {ms}$

### Age and sex differences

Typically, age and sex are investigated as covariates influencing the dynamic response to head and neck perturbations. Hence, we present the experimental results split up into three age groups and two sexes in the following.

**Fig. 3:**
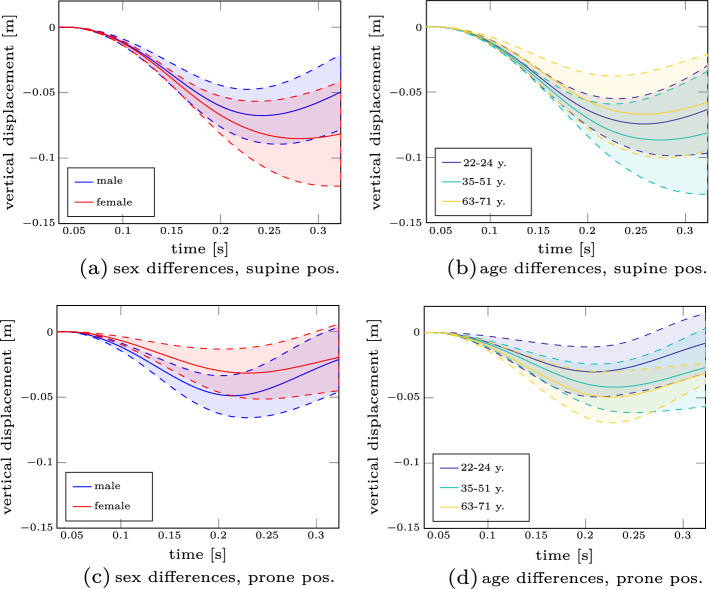
Differences in vertical displacement for different ages and sexes. Experimental vertical displacement trajectories for both the supine (**a**, **b**) and the prone position (**c**, **d**) are shown. The differences of age and sex are highlighted with different colours

The differences for these covariates for the vertical displacement curves are shown in Fig. 3. The panels on the left (Fig. 3a, c) show the trajectories for the different sexes with different colours, the panels on the right (Fig. 3b, d) the ones for the age groups, respectively. Based on these results, we can note two things: first, male participants fall a shorter distance than female participants in the supine position. In the prone position, this behaviour is reversed (not significant, ($p = 0.07$). A detailed overview of the statistical analysis is given in Additional file 1: Table E4 regarding the covariate sex and Additional file 1: Table E5 regarding the covariate age in the supplementary material E). Second, in the supine position, elderly (63–71 years) people fall less strongly compared to younger people, in the prone position, this behaviour is also reversed.

We can observe similar trends dependent on the force direction for the peak acceleration values. An overview of all acceleration values split up into different sex and age groups is given in Table 3. Two main points can be emphasized here: first, for the elderly people both the peak linear and the peak rotational acceleration, as well as the time to peak acceleration, have the smallest value in the supine case. However, this behaviour is reversed in the prone case. Here, both the peak linear and rotational accelerations have the highest values compared to the other age groups. Second, the exact opposite applies to the women participating in this study compared to the male participants. The peak linear and rotational acceleration, as well as the time to peak, is higher in the supine case. In contrast to this, the peak linear and rotational acceleration of female participants is smaller compared to male participants in the prone case.

**Fig. 4:**
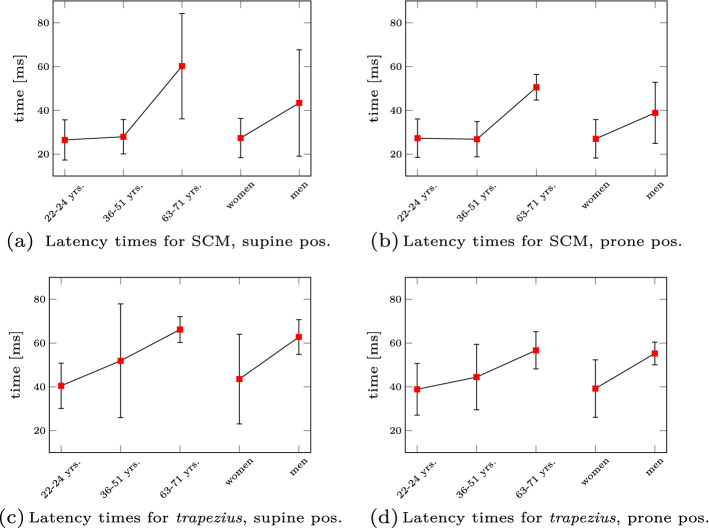
Experimental latency times. The latency times for the *SCM* and *trapezius* muscles in both the supine (**a**, **c**) and prone position (**b**, **d**) are shown. The mean and standard deviation were calculated for different age and sex groups

In contrast to this force-directional dependency, the latency times (the difference between the perturbation and muscle onset) show similar trends for both force directions. They are shown as bar plots in Fig. 4 where the mean value is shown as a red square and the standard deviation as black bars. Based on the age and sex subgroups, we can see that elderly people have higher latency times (mean value is 1.3–2.2 times higher compared to the 36–51 years old, significant for SCM ($p < 0.05$)). Further, we can state that men seem to have higher latency times than women. Even though there might be a slight bias (only men were in the oldest age group), these findings are in accordance with the literature [14, 72].

**Table 3:** Subgroup-specific peak accelerations (given as mean ± standard deviation)

	Age groups			Sex	
	22–24 years	36–51 years	63–71 years	Male	Female
Peak lin. acc., supine	− 0.75 ± 0.1 g	0.79 ± 0.1 g	− 0.66 ± 0.2 g	− 0.71 ± 0.2 g	− 0.77 ± 0.1 g
Time to peak lin. acc., supine	45.8 ± 2.5 ms	43.6 ± 2.9 ms	41.4 ± 2.7 ms	42.2 ± 2.9 ms	45.2 ± 2.7 ms
Peak rot. acc., supine	67.7 ± 6.0 rad/s^2^	64.3 ± 5.6 rad/s^2^	50.3 ± 13.8 rad/s^2^	57.2 ± 13.5 rad/s^2^	66.0 ± 6.2 rad/s^2^
Time to peak rot. acc., supine	52.7 ± 7.6 ms	53.7 ± 13.7 ms	48.8 ± 4.4 ms	49.3 ± 5.9 ms	54.1 ± 11.2 ms
Peak lin. acc., prone	− 0.43 ± 0.3 g	− 0.51 ± 0.2 g	− 0.62 ± 0.2 g	− 0.65 ± 0.2 g	− 0.40 ± 0.2 g
Time to peak lin. acc., prone	36.7 ± 1.9 ms	35.6 ± 2.1 ms	37.0 ± 2.2 ms	36.4 ± 2.6 ms	36.4 ± 1.7 ms
Peak rot. acc., prone	38.7 ± 20.2 rad/s^2^	43.5 ± 13.7 rad/s^2^	53.0 ± 17.4 rad/s^2^	54.7 ± 14.5 rad/s^2^	36.1 ± 15.3 rad/s^2^
Time to peak rot. acc., prone	54.0 ± 10.3 ms	63.8 ± 6.1 ms	55.6 ± 12.7 ms	58.5 ± 9.9 ms	57.4 ± 10.8 ms

### In-depth force analysis

**Fig. 5:**
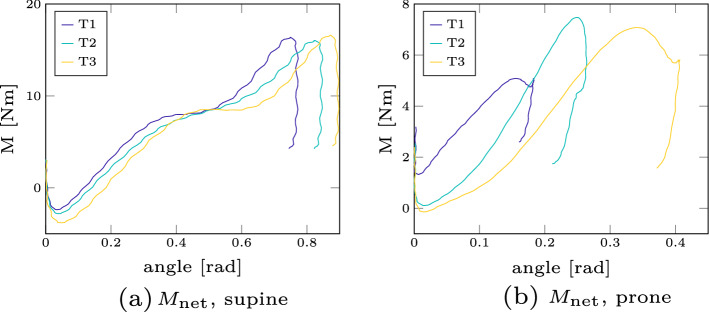
Experimental net moment. The net moment $M_\text {net}$ plotted over the angle is shown here for a representative participant (participant 4), in both experiments (supine and prone position). The different colours represent the three separate trials

As a result of calculating the inverse dynamics, we show the calculated net moment $M_\text {net}$ plotted over the angular displacement for a representative participant (participant 4) in Fig. 5. The three different curves correspond to the separate trials. It can be seen that the slope is almost linear in the beginning, which is comparable to previous studies [54–56]. Furthermore, we see a hysteresis at the end of the torque–displacement curve in our experiments: the torque increases at first for increasing angular displacement, then reaches a maximum torque and displacement value and then gets smaller for decreasing displacement. For an overview of all torque–displacement curves for each participant, we refer to the two figures for the supine and prone case in Additional file 1: Supplementary material A. Based on these figures, one can conclude that the overall curve characteristics are similar within participants and especially in between trials.

**Fig. 6:**
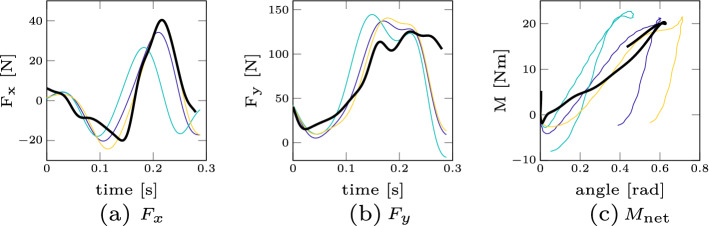
Dynamic quantities. Comparison of different dynamic quantities for both the experimental results (participant 17, all three trials, displayed in colour) and the simulation result (displayed in black)

As a second result, we compare the three different dynamic quantities resulting from Eq. (6) for the experimental as well as the simulated data in Fig. 6. We show the experimental results for participant 17 because both the height and weight have similar values compared to the simulation model. Several points can be noted here: first, the peak force in the vertical direction $F_y$ is more than three times higher than in horizontal direction $F_x$. Second, both the peak forces as well as the general force curve over time are roughly similar in both experiment and simulation. Finally, the simulation model is able to predict the linear increase of moment over angle in the beginning and has a similar peak moment as in the experiments. There are some discriminable differences between the simulation and experimental results, e.g. the hysteresis behaviour at the end of the torque–displacement curve where the decrease in torque for a decreasing displacement is less than in the experiments. However, these differences are less pronounced than the observed variations between participants (e.g. as shown in the torque–angle curves in Additional file 1: supplementary material A of all participants).

### Controller variation in the simulation

**Fig. 7:**
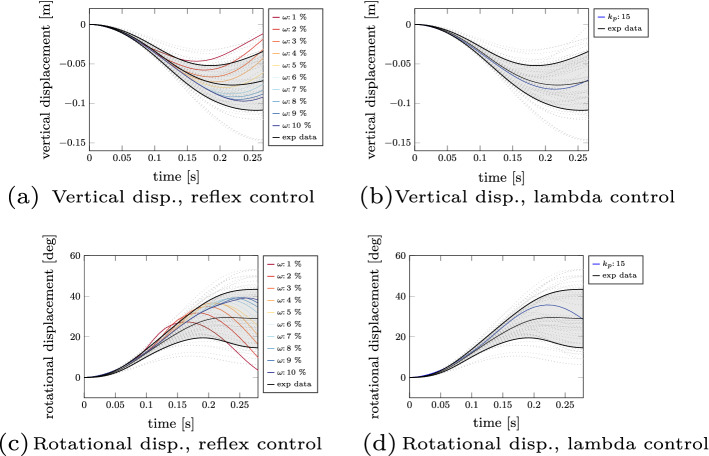
Results of controller variation. Simulation results showing the vertical and rotational displacement trajectories for both the reflex controller (**a**, **c**) and the lambda controller (**b**, **d**). In comparison to the simulation results (displayed in colour), the mean value of the experimental data is shown with a black solid line, the standard deviation of the experimental data is shown as a grey area and all experimental trajectories are shown as dashed grey lines

We varied the reflex control parameter $\omega$ representing the strain threshold to see how this influences the prediction of human reflexive movement. The results for the vertical and rotational displacement are shown in Fig. 7a, c, respectively. Note, that the vertical displacement of Marker 2 from the experiments corresponds to the head centre of gravity of the model as their position are roughly aligned. Based on the figures, we can see that thresholds $\omega$ between $3-10 \%$ fit the experimental corridor well (standard deviation shown as a grey area). The other thresholds predict trajectories which lie outside the standard deviation, but still show similarities to trajectories of real participants (shown as dashed grey lines). Besides, we see that for smaller reflex thresholds the peak displacement is less pronounced, which in turn reduces both the linear and rotational peak accelerations. Furthermore, we can state that the trajectory predicted using the reflex controller with a threshold of $5 \%$ has the smallest L2-error compared to the experimental mean trajectory.

Therefore, we used this threshold for a comparison with the lambda controller and computed comparable $k_p$ values (an exemplary transformation from reflex to lambda control parameters is shown in Additional file 1: supplementary material B). The predicted vertical and rotational displacements using the lambda controller are shown in Fig. 7b, d. It can be seen that the predicted trajectory is close to the experimental mean trajectory.

## Discussion

**Fig. 8:**
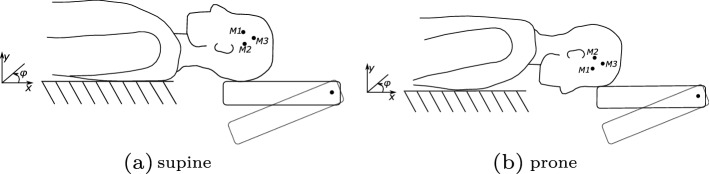
Sketch of the volunteer placement. The volunteer were placed in supine (**a**) and prone (**b**) position. The participant’s head was supported by a trapdoor released at the start of the experiment. The three recorded markers are labelled as M1, M2 and M3 in the figure. Here, $\varphi = 0$ represents the starting position, where the head is at rest

In this study, we characterized the reflexive response to head–neck perturbations using a ’falling heads’ experiment (as shown in Fig. 8). For this purpose, we extracted kinematic (displacements, accelerations), dynamic (stiffness) as well as neuronal (EMG latencies) quantities from the experimental data and compared them both to literature data as well as to our simulation results. We demonstrated that our results fit well with the data found in literature. Furthermore, we showed that a different behaviour for the supine case (extension) compared to the prone case (flexion) is observable: we see less vertical displacement (see Fig. 1), smaller peak accelerations (see Table 1) and a faster response in terms of EMG latency time (see Table 2) in the prone case. The reason for this difference is the muscles’ ability to generate a more significant extension moment compared to flexion, as reported in the literature [74]. This larger extension strength over flexion has two main reasons: first, the postural role of extensor musculature and second, the apparent muscle mass difference between posterior and anterior muscles of the cervical spine [32, 71]. This finding might have direct implications for the evaluation of concussion and whiplash-associated-disorder risks because if the force is applied from a different direction (frontal versus back), the peak linear and rotational accelerations values are reduced as shown in Table 1.

Age and/or sex are typically used to cluster people in groups for a more structured discussion of the results of biomechanical studies. In the present study, the comparison of the results, distinguished by age or sex revealed differences (Figs. 3, 4; Table 3), however most of them were *not* statistically significant (see also Additional file 1: Suppl. Material S5). Therefore, we want to stimulate a broader discussion of how to group people according to the mentioned attributes in general. Traditionally, as was done to analyse the data in this contribution, age groups are defined to account for younger, middle-aged and more elderly people, while sex groups separate women and men. This subgrouping inevitably implies the study results to be dependent on the number of days lived since birth or the biological sex. However, we ask whether this is always appropriate? From an ergonomics perspective, grouping along biological sex and age is appropriate, because it can be easily determined and, thus, is a trivial and valid task. It enables to correlate generic characteristics with the respective groups [69]. Correlation of demographic characteristics such as height and weight with, for example, biological sex also holds for our experimental data (see Table 4). Such a correlation even allows to guess, for example, whether sizes of seats or doors fit most people or a special group of people (e.g. elderly, [35]) or how to design work places [28, 59]. Undoubtedly, ageing affects individual system properties, like muscle torque, velocity and power [39]. In this sense, the classical grouping is appropriate. From a biomechanics standpoint, it is key to understand the underlying cause of experimental observations, e.g. forces or torques. In living systems, these forces or torques depend on the current state of the respective system. For example, how often a ligament has been stretched close to its individual failure strain. In this case, *number of stretches close to failure* and *individual failure parameter* are two important values to determine the ligament’s state. It might turn out that a neck ligament of a 90-year old man is in the same degenerated state relative to its initial state when this very person was 20 years old—say 90% degenerated, compared to a 60-year-old woman who gave birth to five children. So in this hypothetic example, a grouping into age or biological sex does not seem appropriate to understand causes of potentially different head kinematics. Related research fields such as clinical biomechanics have proposed similar ideas, where they suggested to group cervical spines according to biological age, e.g. [75]. They characterize biological age as degeneration of ligaments among other factors rather than relying on chronological age. Another interesting approach comes from the field of computer vision, where they take body shapes into account to create and scale human body models [44]. This overcomes the need to scale digital human body models solely based on height, weight and biological sex. The advent of these digital human body models allows to generate synthetic compositions of humans. Already five decades ago, three-dimensional, mathematical models of the human body emerged [21]. The core idea of representative segments for which individual body parameters are determined based on data regression remained but was improved over time [9, 11, 76, 80]. There have even been attempts to account for age [24, 31]. It seems now is the right time to start understanding individual contributions of degenerated (aged) and subject-specific body parts on functional characteristics like joint angle progression. Therefore, we would like to encourage research towards finding new concepts to distinguish humans based on causal dependencies of forces and torques around joints and age (degeneration). As we have stated above, this paragraph intends to stimulate the discussion. We see a need to find more appropriate grouping, but unfortunately have no solution yet. However, we hypothesize to group people and scale human body models according to attributes such as, e.g. body shape, degeneration, and fitness, among others. Therefore, we conclude that current grouping might be inappropriate and attenuate our findings with respect to the subgroups presented in this study (see Sect. 2.2).

Experimental validation of active Human Body models (AHBMs) raises significant challenges as one needs to validate both the human body’s passive and active mechanical characteristics and its subsystems. To ensure this validation process, various studies are focusing both on the whole body [12, 15, 27] and subsystems [8]. However, there is a clear need to explore the passive and active behaviour of the neck region required [64]. Our study offers force (Fig. 5) and dynamic (Fig. 6) data from the experiment and simulation for this specific region. A recent work where such force data were directly used to improve a digital human body model is the study of Mörl et al. [51]. Here, they demonstrated how a similar stiffness calculation could be used directly to adjust their lumbar spine model values taken from well-established literature sources of ligament and passive muscle stiffness to fit the experimentally measured stiffness. Similarly, researchers could take our data to improve their neck models. Furthermore, our data set could be used to validate and compare existing control strategies. In our model, we only used two stretch-based reflex controllers, however, recently a more sophisticated control approach for a multi-body head–neck model was proposed by Zheng et al. [82] where the vestibular reflex was additionally included. This reflex is modulated by sensing the disturbed head motion (linear acceleration and angular velocity) by the vestibular organs in the inner ear (both the semicircular canals and the otoliths) [36]. Therefore, the main purpose of the dataset presented here (available with open-source access), is to serve as a benchmark test for both passive neck properties, but also for different muscle control strategies in the same model and its implementations in various codes. We believe that the outcome and the experimental data of this study will help to improve existing and to develop potentially better AHBMs.

Initially, we posed the question how the biomechanical reflex response changes, if we vary the neuronal state in the simulation. Therefore, two muscle length feedback controllers were used to run a ’falling heads’ simulation with varied controller variables (threshold $\omega$ and spindle feedback gain $k_p$, respectively) to represent the sensitivity of the neuronal state to the perturbation. Based on these simulations, we postulate that we are able to synthesize biophysically valid human movement with our control approach (as shown in Fig. 7). This is in line with the literature, where various authors [26, 57, 58] showed that including muscle activations helps to improve the agreement of experimental and simulated responses by decreasing the acceleration of the head. Furthermore, we showed that we are able to modulate the response using simple control parameter adjustments (see Fig. 7a, c). On the one hand, this modulation of the response can be used to represent a large variability of participants. On the other hand, it shows how a higher sensitivity of the neuronal state (in term of reflex thresholds) helps in reducing acceleration peaks. Whether these reflex thresholds are set explicitly like this by the nervous system as a result of an optimization function for unexpected perturbations, e.g. to minimize these accelerations peaks, stress or in general injury risks, was not investigated in this study. However, previous work showed that by using optimization principles it is possible to explain and predict voluntary movement, e.g. for walking [1, 50], eye movements [22], standing from a chair [53], and point-to-manifold reaching [78]. Whether such an optimization function is also adapted for unexpected perturbations, should be explored in further work. Independent of which optimization criterion is used, future work can directly exploit the correlation between a reduction in peak acceleration and the sensitivity of the neuronal state to develop better injury prevention strategies. This means that if mechanisms are applied to prepare humans to upcoming events (e.g. by sound signals), they can pre-tune their reflex gains accordingly, which in turn might reduce potential injury risks.

## Conclusions

In this study, we present novel experimental data in a ’falling heads’ setup in order to investigate individual reflexive responses to head–neck perturbations. We extracted several biomechanical parameters such as joint stiffness, peak acceleration and latency times based on this data.

Analysing this data, we show that there is a large difference in the individual reflexive responses between participants, e.g. for the peak falling height. Furthermore, we show that the perturbation direction has a significant influence on the kinematic quantities (e.g. peak linear and rotational acceleration) which is not reflected in the EMG latency times. Finally, we show that the musculoskeletal simulations with a reflex controller provide comparable results to the experiments. The setup of these numerical simulations is simple, making them an ideal candidate for future validation requirements in virtual testing procedures.

Concluding, a novel experimental dataset for head–neck perturbations including two different force directions (flexion and extension) for a larger number of healthy participants with different ages and sexes) is now available open-source. This experimental dataset can be used as a benchmark test to improve, compare and develop better human body models and muscle control strategies.

## Methods

We conducted experiments in which relaxed volunteers were placed on a table in a supine and a prone position to investigate the individual responses to head–neck perturbations. The subject’s head was supported by a trapdoor, which was suddenly released. This action resulted in a free-fall movement of the head until the subject reacted to the perturbation by developing a force in the antagonistic muscles, leading to the deceleration of the falling head. We recorded the kinematic trajectory of the head and the electromyographic (EMG) signal of the *sternocleidomastoideus* and the *trapezius* muscles to investigate this reflexive behaviour. Furthermore, we performed simulations matching the supine experiments using the academic THUMSv5 model [33] including Hill-type muscles activated using two different threshold-based reflex controllers. For both scenarios (real-world experiment and simulation), we performed an inverse dynamics analysis to determine the underlying force interactions, give an estimate for the stiffness values and use this data to validate the used human body model. The methods are described in further detail in the following.

### Participants

Seventeen subjects volunteered to participate in the experiment (7 males, with an age range of 22–71 years). All of them were healthy. The demographic characteristics of all participants are given in Table 4. To investigate whether age or sex influenced the reflexive behaviour in this experimental setup, we divided the experimental data for some of the analysis into three age groups and two sexes (male, female) as shown in Table 4. Written informed consent was obtained from each participant in the study, which was approved by the ethics committee of the Karl-Franzens-University of Graz (reference number: 39/67/63 ex 2014/15). In addition, we certify that all methods were carried out in accordance with all applicable institutional and governmental regulations concerning the ethical use of human volunteers and in accordance with the Helsinki declaration during the course of this research.

**Table 4:** Demographic characteristics for participants ($n = 17$), given as mean ± standard deviation

	Age groups			Sex	
	22–24 years	36–51 years	63–71 years	Male	Female
	($n = 7$)	($n = 6$)	($n = 4$)	($n=7$)	($n=10$)
Age [years]	$22.4\pm 0.8$	$44.7 \pm 6.4$	$66.5 \pm 3.4$	$51.9 \pm 20.7$	$32.8 \pm 11.9$
Weights [kg]	$65.6 \pm 9.5$	$60.0 \pm 11.6$	$68.5 \pm 8.4$	$72.0 \pm 8.6$	$58.9 \pm 7.5$
Height [m]	$1.70 \pm 0.06$	$1.69 \pm 0.08$	$1.78 \pm 0.01$	$1.78 \pm 0.03$	$1.67 \pm 0.03$
BMI [kg/m^2^]	$22.6 \pm 3.3$	$20.9 \pm 2.1$	$21.8 \pm 2.8$	$22.6 \pm 2.5$	$21.3 \pm 2.9$
Sex [#m, #f]	2 m, 5 f	1 m, 5 f	4 m, 0 f	7 m, 0 f	0 m, 10 f

### ‘Falling heads’ experiment

An illustration of the conducted ’falling heads’ experiments is shown in Fig. 8. Participants were placed on a table first in the supine and then the prone position. The head was supported by a trap door, which was unexpectedly released by an electromagnet at irregular intervals. In the supine position, it was ensured that the T1 vertebrae was placed directly on the edge of the table. They were not restrained, however, in the supine position, arms were placed on the abdomen of the participants and in the prone position, arms were placed such that they hang quietly in an angle of $90^{\circ}$ to the horizontal line (armpits directly on the table’s edge). Furthermore, the boundary conditions were adapted to account for the different anthropometries of the participants by manipulating the position and height of the table to ensure two things: first, that the edge of the table was parallel and on the same height as the trapdoor. Secondly, to adjust the gap between the table and the trapdoor such that head was always placed on the same marked position on the foam of the trapdoor. In the prone position, this position was equivalent to placing the forehead to nasal bone on the trapdoor. The trapdoor was not moved due to calibration reasons. For every participant, the experiment was repeated three times in each position. The free fall would gently brake by the cushioned trapdoor after a maximum angle deviation of $40^{\circ }$ to avoid any injuries (if the participants did not react before that). The subjects were encouraged to relax between each drop. Relaxation was also checked based on the level of EMG activity. To ensure that the recorded kinematics and the EMG signal are synchronized, we included a hardware trigger (by breaking the power circuit) which releases the trapdoor, starts the recording of the camera and sets a trigger index in the EMG signal.

### Kinematic analysis

Head and neck kinematics were recorded using a HCC-1000 camera and HCC Control software (VDS Vosskühler GmbH/Germany) at a sampling rate of $462\,\hbox {fps}$. Three markers were recorded to detect both translational as well as rotational movements of the head. Volunteers were asked to wear a swimming cap in order to place the markers better and to avoid that they are obscured by hair. Marker 1 was positioned close to the eyes on the *sphenoid* bone. Marker 2 was positioned in sight line with a distance of $4\,\hbox {cm}$ to Marker 1 and corresponds to the head centre of gravity projection in the sagittal plane. Finally, Marker 3 was attached such that all three markers formed an equilateral triangle as shown in the sketch in Fig. 8. The motion analyses were performed with custom software written in Matlab^®^ (Mathworks, Natick, MA, USA) based on the recorded marker positions.^2^ From these recorded trajectories, we calculated displacements, velocities and accelerations and processed the signals with a Butterworth low-pass filter (cut-off frequency 15 Hz; fourth-order).

### Electromyographic analysis

Muscle activation was monitored using surface EMG of the *sternocleidomastoideus (SCM)* muscle and the *trapezius* muscle. The EMG activity was recorded at $1000\,\hbox {Hz}$ using myoResearch software (Noraxon/USA). The placement of the electrodes at the *trapezius* muscle was done according to SENIAM guidelines [23]: the electrode pair was placed in the middle of the line between the spinous process of the 7th cervical vertebra and the acromion. The electrodes for the *sternocleidomastoideus (SCM)* muscle were placed over the middle part of the SCM muscle according to Sheykholeslami et al. [65]. All electrodes were attached parallel to the muscle fibre orientation at a distance of 20 mm. The reference electrode was fixed on the acromion. Volunteers were asked to relax their muscles before the onset of the movement. To measure the time delay between the release of the trapdoor and the first muscle activation, we used a threshold-based EMG onset detection method [43], which is a combination of two other methods [4, 25]. The main idea is to use the full-wave rectified EMG signal $y_k$ to compute a test function $g_k$ and to define the muscle activity onset $t_0$ as the point of time when this test function exceeds a threshold value. This threshold value is specified as a multiple *h* of standard deviations. As a rule of the algorithm, at least *T*1 samples should be higher than the threshold value, while allowing for *T*2 samples to fall below this value. The algorithm is summarized below [68]:

1 $$\begin{aligned} t_0= & {} \min _{k\le W}(g_k\le h)-W+1, \end{aligned}$$

2 $$\begin{aligned} g_k= & {} \frac{1}{{\hat{\sigma }}_0}\left( \frac{1}{W}\sum _{i=k-W+1}^{k}y_k-{\hat{\mu }}_0\right) . \end{aligned}$$

Here, *W* denotes the width of the fixed-size sliding test window, *k* the current time, and ${\hat{\mu }}_0$ and ${\hat{\sigma }}_0$ the mean and standard deviation of the *M* initial samples of $y_k$, respectively. Five parameters need to be chosen for the onset detection: the number of initial samples *M*, the number of multiple standard deviations to calculate the threshold *h*, the window size *W*, the minimum number of samples above the threshold *T*1 and the number of samples which are allowed to fall below the threshold in this period, *T*2. Detected latency times $t_0$ smaller than $10\,\hbox {ms}$ or larger than $100\,\hbox {ms}$ were considered as invalid. The lower value was chosen because due to the conduction times of the action potential propagation and the central delay of the involved synapses [41], the muscles cannot be activated instantaneously. The upper value was chosen because we are only interested in the reflex activation and all latency times larger than $100\,\hbox {ms}$ were considered to be a voluntary reaction. We justify this restriction interval because previous studies found neck muscle latencies in response to perturbations to be in the range of 18.6–$88\,\hbox {ms}$ [7, 14, 29, 30, 47, 63, 72].

An optimization algorithm with two objective criteria was utilized to find the five necessary parameters for the neck reflex latency. The first criterion ensures that the optimized parameters minimize the number of invalid latency times $t_0$. Additionally, the second criterion tries to find parameters which minimize the standard deviation $\sigma$ of latency times between trials for each subject. Such an approach allowed us to find parameter values as objectively as possible without having to rely on expert opinions.

We define the entire objective criterion $\varepsilon$ as:

3 $$\begin{aligned} \varepsilon _1= \sum _{i=1}^{n}\sum _{j=1}^{m} (t_0(i,j) < 10) \vee (t_0(i,j) > 100), \end{aligned}$$

4 $$\begin{aligned} \varepsilon _2= \sum _{i=1}^{n} \sigma (t_0(i,j)),\, \text {with } j = 1 \dots m, \end{aligned}$$

5 $$\begin{aligned} \varepsilon= w \cdot \varepsilon _1 + \varepsilon _2. \end{aligned}$$

Here, *n* denotes the number of subjects (in our case $n=17$) and *m* the number of trials (in our case $m=3$). We chose a weighting factor $w = 100$ to emphasize the importance of detecting as many valid latency times as possible. The objective function was minimized using the *surrogateopt* algorithm in Matlab^®^ (Mathworks, Natick, MA, USA).

### Inverse dynamics analysis

We formulate the equations of motions for the head–neck segment to extract the joint torque $M_\text {net}$ exerted on the head–neck segment by the trunk segment at the connecting joint. Previous studies modelled the head–neck system as a rigid inverted pendulum with a fixed centre of rotation [55, 67]. For our analysis, we had to extend their approach because the investigated head movement in our study is a combination of translation and rotation. For the rigid head–neck segment as part of an open chain in the sagittal *x*-*y*-plane, the following three equations of motion apply [17, 76]:

6 $$\begin{aligned} F_{x}= m\ddot{x}, \end{aligned}$$

7 $$\begin{aligned} F_{y}= m(\ddot{y}-g), \end{aligned}$$

8 $$\begin{aligned} M_\text {net}= I \ddot{\varphi } - r_{x}F_{y} + r_{y}F_{x}. \end{aligned}$$

The force $F_y$ acting in the vertical axis is dependent on the gravitational acceleration *g*. We estimate the head–neck mass *m* based on [76] as $8.1\%$ of the total subject’s mass. We calculate the moment of inertia *I* about the centre of mass (based on data from [76]) and scaled accordingly with the total subject’s mass and height. The vectors $r_x$ and $r_y$ represent the distance between the bone centre of mass (COM) to the centre of joint rotation. The perturbing force ($m\cdot g$) was delayed by $4\,\hbox {ms}$ to take into account that the detachment of the head from the trapdoor does not happen instantaneously (alternatively a contact force at $t=0$ could be defined). This delay was determined by comparison of the onset of the perturbing force and the measured head acceleration traces and adjusting the delay accordingly. We use this model and the calculated torque to estimate neck stiffness for the first $150\,\hbox {ms}$ following perturbation onset similar to the study of Simoneau et al. [67]. This stiffness is called effective neck stiffness because it represents a combination of intrinsic and reflexive components. We calculate this stiffness for the first $150\,\hbox {ms}$ as a linear approximation, i.e. the change in torque versus change in angle [56]:

9 $$\begin{aligned} S = \frac{\Delta M_\text {net}}{\Delta \varphi }. \end{aligned}$$

Note that there are various approaches to calculate joint stiffness. Many of them are more complicated comparing to the proposed one. For example, one can include initial rest angle, damping, shifts of rest length and nonlinearities to calculate leg stiffness [6, 16]. Such methods allow making statements about the underlying biomechanical structures. However, we chose this somewhat reduced approach to enable a comparison of our absolute values to similar neck stiffness calculations done by Simoneau et al. [67] and Portero et al. [56].

### Simulations

We compared the experiments of the supine case to simulations of the head-fall setup. There are two main computational methods used for simulations with human body models: Multibody (MB) Dynamics and Finite Element (FE) Analysis and the current study utilizes the latter. Among the most advanced FE Active Human Body Models (AHBMs) with muscle elements and a controller integrated into the whole body we can name the Global Human Body Models Consortium (GHBMC) [10], Total HUman Model for Safety (THUMS) [33, 34], SAFER A-HBM [40], THUMS TUC-VW AHBM [70, 79] and the AHBM developed during the joint collaboration of Mercedes-Benz AG and University of Stuttgart [49, 52]. A detailed comparison of these models and muscle control strategies used is given in Additional file 1: Table D2 in the supplementary material D. For our simulations, we used the THUMS v5 AM50 Occupant Model Academic Version [33] using the FE simulation software LS-DYNA. This model is driven by Hill-type muscles which are activated using two different threshold-based stretch reflex controllers. Please note, that there exist more sophisticated controllers (e.g. [82] for a MB model) which account also for the vestibular reflex which is modulated due to linear acceleration and angular velocity. Our model modifications and the necessary repositioning to replicate the experimental setup, are described in the following.

#### Positioning of the model

The model was repositioned according to the experimental setup shown in Fig. 8. All the parts, not related to the head, neck or torso regions were removed. Besides, translational and rotational constraints were introduced to the pelvis and abdomen. The gravitational load was applied according to the new model orientation. The table was implemented using a planar rigid wall supporting the lower back of the model. Furthermore, a second rigid plane was implemented to model the trapdoor supporting the head–neck complex. To ensure that the model starts in a planar equilibrium, we let the model settle due to the gravitational acceleration. Then, after this pre-simulation, the model was considered identical to the initial position of the volunteers and the second rigid plane was released to simulate the release of the trapdoor.

#### Modification of the muscles

In the standard version of the THUMS v5 model, muscles are modelled with a material named *MAT_MUSCLE (*MAT_156) [46]. Recently, a new Hill-type muscle material with a more realistic eccentric force–velocity relation and serial damping [18, 20] was implemented in LS-DYNA as a user-defined material which was named the extended Hill-type muscle model (EHTM) [37, 38, 48]. It shows a better material model accuracy compared to the standard muscle model *MAT_MUSCLE (*MAT_156) used in LS-DYNA [37, 77]. Therefore, in order to make the THUMS v5 more biophysically valid, we replaced the *MAT_MUSCLE material by this user-defined EHTM material for all muscles defined in the head–neck region (see Appendix A of THUMS v5 documentation [73]). Corresponding *MAT_MUSCLE parameters were converted into the EHTM parameters according to the procedure described in the supplementary material C. Note, that such a conversion is not unambiguous because the new muscle material requires at least one additional parameter, e.g. the ratio between the optimal fibre length $l_\text {opt}$ or the tendon slack length $l_\text {SEE,0}$ (can be taken from literature). Similar to [62], we define this ratio as:

10 $$\begin{aligned} m_\text {ratio} = \frac{l_\text {opt}}{l_\text {opt}+ l_\text {SEE,0}}. \end{aligned}$$

Using the assumption that the original muscle length $l_0 = l_\text {opt} + l_\text {SEE,0}$, we can then directly calculate the missing values of the model (abbreviated with mdl) based on the literature data (abbreviated with lit):

11 $$\begin{aligned} l_\text {opt, mdl}= m_\text {ratio,lit}\cdot l_\text {0,mdl}, \end{aligned}$$

12 $$\begin{aligned} l_\text {SEE,0,mdl}= l_\text {0,mdl}-l_\text {opt, mdl}. \end{aligned}$$

This a valid assumption for a normal upright state [62], which we have for the head–neck muscles. It should be mentioned here, that in the special case of $m_\text {ratio} = 1$, we directly set the tendon slack length $l_\text {SEE,0}$ to $1\,\hbox {mm}$. As literature source for $m_\text {ratio}$, we used values taken from [5]. The neck muscle parameters we used in this study are provided open-source.^3^ Note, that we excluded the *digastricus*, the *mylohyoideus* and the *stylohyoideus* muscles from the simulation model. This was done because they are mainly responsible for lifting the jaw or tongue, which is not relevant for the investigated movement in this study. Besides, the specific parameters for these muscles were not available in Borst et al. [5].

#### Muscle reflex controller

A dedicated reflex controller was proposed and included in the user-defined EHTM material model to determine the muscle stimulation $u_i$ based on the current strain [13, 38, 48]. The latest improved open-source code version is made available.^4^ The proposed reflex controller is a stretch-based muscle length controller, which activates every *i*th muscles with $100 \%$ stimulation $u_i$ as soon as a particular strain threshold $\omega$ is exceeded. The detailed description of the controller’s logic is given in Table 5 (adapted from [38]). Three parameters can be defined prior to the simulation: the delay time $\tau$, the reference length of the contractile element $l_\text {CE,ref}$ and the threshold $\omega$. For the ’falling heads’ setup, we chose the initial lengths of the contractile element after settling on the table as reference lengths $l_\text {CE,ref}$ because they correspond to the relaxed state of the participants lying on the table. Previous studies [13, 26] reported to use a delay value of $25\,\hbox {ms}$, which is why we chose this value for $\tau$. The threshold $\omega$ was varied between 1 and $10 \%$ because this range has a good agreement (small L2-error) with the experimental data (see also Fig. 7). As we show later in the Results section, the reflex threshold $5 \%$ has the smallest L2-error compared to the mean of the experimental data. Therefore, the simulation results are shown only for this curve if not stated otherwise.

**Table 5:**
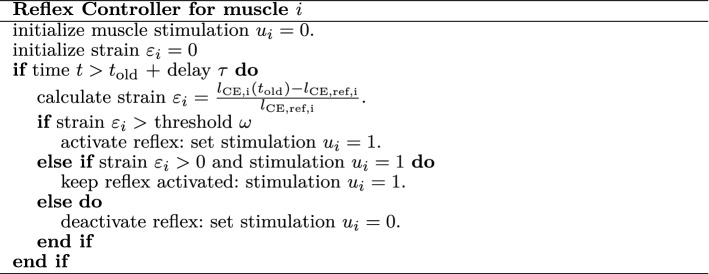
Reflex controller algorithm (adapted from [38])

#### Muscle lambda controller

As an alternative to the reflex controller, there also exists a neural feedback controller [2] based on the muscle fibre length of the contractile element (CE) $l_i^\text {CE}$. The stimulation signal $u_i^\text {lambda}$ is calculated as follows:

13 $$\begin{aligned} u_i^\text {lambda} := \frac{k_p}{l^\text {CE,opt}}(l_i^\text {CE}(t) - \lambda _i). \end{aligned}$$

With this, a sensory feedback mechanism can be described, because $u_i^\text {lambda}$ depends on the difference between the currently desired fibre length of the contractile element $\lambda _i$ and the actual CE fibre length $l_i^\text {CE}$. The difference is weighted by a muscle spindle feedback gain $k_p$ and the optimal CE fibre length $l^\text {CE,opt}$.

## Supplementary Information

**Additional file 1: S1 Appendix**. Inverse Dynamics Analysis: Moment of all participants. We show two figures containing the resulting moment of the inverse dynamics analysis for all participants for the supine and prone case.** S2 Appendix**. Transformation between muscle reflex and lambda controller. We present a more detailed explanation how the parameters between the reflex and lambda controller can be transformed.** S3 Appendix**. A guideline for parameters conversion from *MAT_MUSCLE to the EHTM. We give a more detailed guideline how the material parameters from the LS-DYNA *MAT_MUSCLE material can be converted to parameters for the Extended Hill-Type Material used in this study.** S4 Appendix**. Overview of Finite Element Active Human Body Models. A detailed comparison of the currently existing FE Active Human Body Models and their muscle control strategies is given in this table.** S5 Appendix**. Statistical analysis of the experimental data. A detailed statistical analysis based on the t-test with all p-values for comparing the three covariates force-direction, biological sex, and age is given.

## Data Availability

The experimental data sets are available at 10.18419/darus-1038 (EMG data) and 10.18419/darus-1132 (trajectory data). The postprocessing scripts for the kinematic and the inverse dynamics analysis are available at 10.18419/darus-2526. The neck muscle parameters we used in this study are also provided open-source: They are available at 10.18419/darus-1145. The improved code of the muscle material (EHTM) is available at 10.18419/darus-1144.

## Abbreviations

*AHBM*: Active human body model
*CE*: Contractile element
*EHTM*: Extended Hill-type muscle model
*EMG*: Electromyographic
*FE*: Finite element
*GHBMC*: Global human body consortium
*MB*: Multibody
*SCM*: Sternocleidomastoideus
*THUMS*: Total human model for safety

## Footnotes

1 The dataset for the electromyographic (EMG) data is available at 10.18419/darus-1038 and the dataset for the kinematic trajectories can be found at 10.18419/darus-1132.

2 The postprocessing code is available at 10.18419/darus-2526.

3 The neck muscle data set is available at 10.18419/darus-1145.

4 The EHTM code and manual are available at 10.18419/darus-1144.
