# Supplementary material for: ‘Falling heads’: investigating reflexive responses to head–neck perturbations
Source: Biomed Eng Online. 2022 Apr 16;21:25. doi: 10.1186/s12938-022-00994-9 (PMC9013062; doi:10.1186/s12938-022-00994-9)
Supplement: Supplementary file 1 — Additional file 1: S1 Appendix. Inverse Dynamics Analysis: Moment of all participants. We show two figures containing the resulting moment of the inverse dynamics analysis for all participants for the supine and prone case. S2 Appendix. Transformation between muscle reflex and lambda controller. We present a more detailed explanation how the parameters between the reflex and lambda controller can be transformed. S3 Appendix. A guideline for parameters conversion from *MAT_MUSCLE to the EHTM. We give a more detailed guideline how the material parameters from the LS-DYNA *MAT_MUSCLE material can be converted to parameters for the Extended Hill-Type Material used in this study. S4 Appendix. Overview of Finite Element Active Human Body Models. A detailed comparison of the currently existing FE Active Human Body Models and their muscle control strategies is given in this table. S5 Appendix. Statistical analysis of the experimental data. A detailed statistical analysis based on the t-test with all p-values for comparing the three covariates force-direction, biological sex, and age is given. [file 12938_2022_994_MOESM1_ESM.pdf]

'Falling Heads': investigating reflexive responses to head-neck perturbations

Isabell Wochner[1*], Lennart V. Nölle[1], Oleksandr V. Martynenko[1] and Syn Schmitt[1]

[1]Institute for Modelling and Simulation of Biomechanical Systems, Stuttgart Center for Simulation Science, University of Stuttgart, Germany.

*Corresponding author. E-mail: isabell.wochner@simtech.uni-stuttgart.de

Appendix A Inverse Dynamics Analysis: Moment of all participants

In the following, we present the resulting moment of the inverse dynamic analysis plotted over the angle displacement for all participants, for the supine case (Fig. A1) and the prone case (Fig. A2).

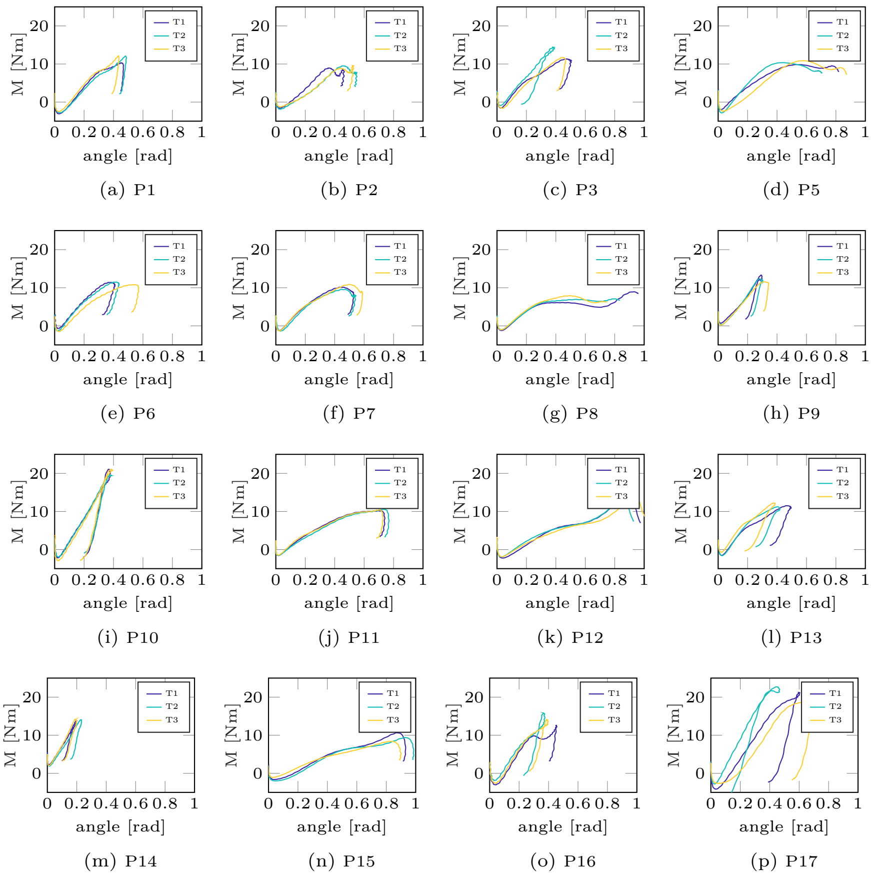

Fig. A1 Comparison of all moments M_{net} plotted over angle for all participants and all trials for the supine experiment.

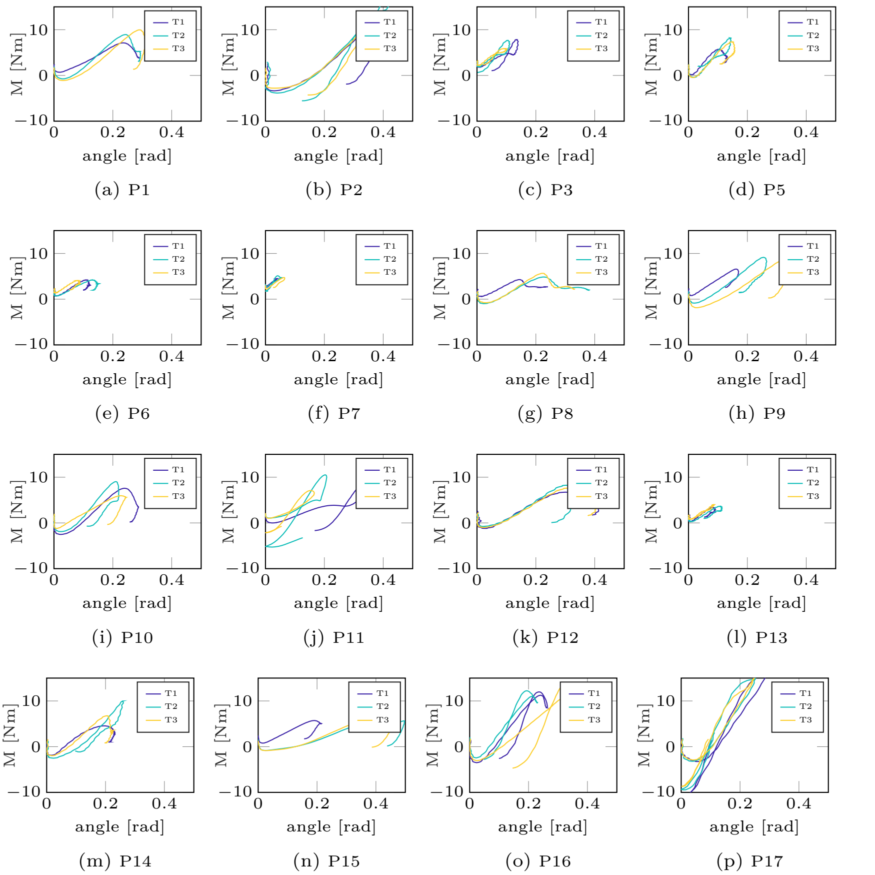

Fig. A2 Comparison of all moments M_{net} plotted over angle for all participants and all trials for the prone experiment.

Appendix B Transformation between muscle reflex and lambda controllers

The previously presented muscle controllers are closely related since they are both dependent on the stretch of the muscle. However, the muscle reflex controller outputs binary values (0 and 1) only, whereas the lambda controller outputs values dependent on the sensitivity of the spindle feedback gain k_p. As soon as the strain ϵ_i surpasses the threshold value ω (at $t = t_{\text{reflex}}$), the muscle is activated with 100% signal by the reflex controller. In this case, we can calculate the necessary k_p to compare the lambda with the reflex controller:

$$1 = \frac{k_p}{l^{\text{CE,opt}}}(l_i^{\text{CE}}(t_{\text{reflex}}) - \lambda_i) \tag{B1}$$

$$k_p = 1 \cdot \frac{l^{\text{CE,opt}}}{l_i^{\text{CE}}(t_{\text{reflex}}) - \lambda_i} \tag{B2}$$

The values of the right hand side of Eq. B2, can be either given directly ($l^{\text{CE,opt}}$) or taken from a simulation with the reflex controller (λ and $l^{\text{CE}}(t_{\text{reflex}})$) but are all known for the transformation. Exemplarily, we calculate k_p for the *sternocleidomastoideus* muscle with a threshold ω of 5% (see values in Table B1):

$$k_p = \frac{168.96\,\text{mm}}{179.74\,\text{mm} - 168.84\,\text{mm}} \approx 15.49 \tag{B3}$$

Table B1 Values of *sternocleidomastoideus,R1* muscle at time $t = t_{\text{reflex}}$ for a reflex controller simulation with threshold $\omega = 5\%$

Variables	Values
$l^{\text{CE,opt}}$	168.96 mm
λ	168.84 mm
$l^{\text{CE}}(t_{\text{reflex}})$	179.74 mm

Appendix C A guideline for parameters conversion from *MAT_MUSCLE to the EHTM

At present, FE software LS-DYNA includes the Hill-type muscle material model *MAT_MUSCLE (*MAT_156) (LSTC, 2016b). This keyword includes variables which differ from the input required for the Extended Hill-Type muscle material (Kleinbach et al, 2017; Martynenko et al, 2018; Kleinbach, 2019). Therefore one needs to perform conversion of material parameters to perform simulations with the muscle elements already existing in the AHBMs. This short guide explains how to make such a conversion.
The EHTM has two sets of muscle parameters:

1. *generic*, which are considered generally the same for a certain group of implemented muscles, and are described in the EHTM user manual (Nölle et al, 2022),
2. *specific*, which include F_{max}, $\Delta F_{\mathrm{SEE},0}$, l_{opt} and $l_{\mathrm{SEE},0}$ and must be determined for each individual muscle by the methods described in the following.

The maximum isometric force F_{max} can be calculated as:

$$F_{\mathrm{max}} = \sigma_{\mathrm{max}} \cdot A, \tag{C4}$$

where σ_{max} is a muscles peak isometric stress and A is its physiological cross-sectional area. Both of these values can be extracted from an original LS-DYNA model as σ_{max} corresponds to the PIS variable defined in the *MAT_156 keyword and A to the variable of the same name in the *SECTION_BEAM keyword (LSTC, 2016a,b).
The force at the nonlinear-linear transition $\Delta F_{\mathrm{SEE},0}$ is given according to Bayer et al (2017) as:

$$\Delta F_{\mathrm{SEE},0} = 0.4 \cdot F_{\mathrm{max}}. \tag{C5}$$

The original muscle length l_0, the optimal fibre length l_{opt} and the tendon slack length $l_{\mathrm{SEE},0}$ are assumed as being interdependent through the relation $l_0 = l_{\mathrm{opt}} + l_{\mathrm{SEE},0}$ as discussed in Chapter Modification of the muscles in the main manuscript. This means that if either l_{opt} or $l_{\mathrm{SEE},0}$ can be determined from literature sources, the missing length parameter can be calculated accordingly. If literature values for both l_{opt} and $l_{\mathrm{SEE},0}$ are available but the given lengths are inconsistent such that $l_{\mathrm{opt,lit}} + l_{\mathrm{SEE},0,\mathrm{lit}} \neq l_{0,\mathrm{mdl}}$, then scaling of both parameters is done according to Eq. (11) and (12) in the main manuscript.
In some cases, larger muscle bodies are subdivided into different muscle strands to better represent their shape under the constraint of only using one-dimensional Hill-type muscle elements. The length scaling methods outlined

previously are only viable, if the number of muscle strands found in literature n_{lit} is equal to the one present in the model n_{mdl}. If $n_{\text{lit}} \neq n_{\text{mdl}}$, then $m_{\text{ratio,mdl}}$ is scaled via one of two different methods depending on the difference in ratio Δm_{ratio} between the maximum and minimum ratios found in literature $m_{\text{ratio,lit,max}}$ and $m_{\text{ratio,lit,min}}$:

$$\Delta m_{\text{ratio}} = m_{\text{ratio,lit,max}} - m_{\text{ratio,lit,min}} \tag{C6}$$

If $\Delta m_{\text{ratio}} < 0.15$ then the scaling of $m_{\text{ratio,mdl}}$ is done through median calculation according to Eq. C7 with $l_{\text{opt,mdl}}$ resulting from Eq. C8.

$$m_{\text{med}} = \frac{1}{n_{\text{lit}}} \sum_{j=1}^{n_{\text{lit}}} m_{\text{ratio,lit,j}} \tag{C7}$$

$$l_{\text{opt,mdl,i}} = m_{\text{ratio,mdl,med}} \cdot l_{\text{0,mdl,i}} \tag{C8}$$

with $1 \leq i \leq n_{\text{mdl}}$.

If $\Delta m_{\text{ratio}} \geq 0.15$ then $m_{\text{ratio,mdl}}$ is instead scaled through linear interpolation according to Eq. C10 while $l_{\text{opt,mdl}}$ is calculated with Eq. C11.

$$m_{\text{slope}} = \frac{m_{\text{ratio,lit,min}} - m_{\text{ratio,lit,max}}}{l_{\text{0,mdl,max}} - l_{\text{0,mdl,min}}} \tag{C9}$$

$$m_{\text{intrp,i}} = m_{\text{slope}} \cdot l_{\text{0,mdl,i}} - m_{\text{slope}} \cdot l_{\text{0,mdl,min}} + m_{\text{ratio,lit,max}} \tag{C10}$$

$$l_{\text{opt,mdl,i}} = m_{\text{intrp,i}} \cdot l_{\text{0,mdl,i}} \tag{C11}$$

No matter which scaling method for $m_{\text{ratio,mdl}}$ has been chosen, $l_{\text{SEE,0,mdl}}$ can always be calculated analogously to Eq. (12) of the main manuscript with Eq. C12.

$$l_{\text{SEE,0,mdl,i}} = l_{\text{0,mdl,i}} - l_{\text{opt,mdl,i}} \tag{C12}$$

Appendix D Overview of Finite Element Active Human Body Models

Table D2 Overview of Finite Element Active Human Body Models

	SAFER A-HBM	THUMS v5,v6	THUMS TUC-VW AHBM	GHBMC M50-OS	A-THUMS-D
**Controller type**	PID	PID	PID	PID	PD
**Control approach**	APF, MLF, STP, CC	APF, CFF, PC	MLF, CC	APF, PC	MLF, CC, PC
**Published in**	Larsson et al (2019)	Kato et al (2017)<br>Kato et al (2018)	Yigit (2018)<br>Sugiyama et al (2018)	Devane et al (2019)	Öztürk et al (2019)<br>Martynenko et al (2019)
**Appearance**	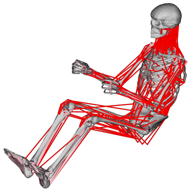	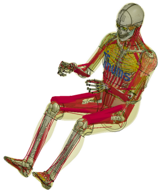	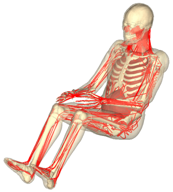	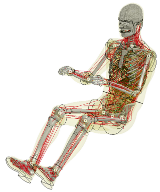	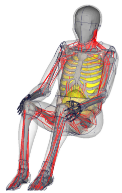

Where:

APF – Angular Position Feedback
MLF – Muscle Length Feedback
STP – Spatial Tuning Patterns
CFF – Contact Forces Feedback
CC – Muscle co-contraction influence
PC – Percentage contribution of each muscle

Appendix E Statistical Analysis of the experimental data

To show whether the differences of the experimental data are significant with respect to the positional disturbance (supine or prone case), age or sex, we performed a statistical analysis based on the t-test (two-tailed). The null hypothesis is that the compared data comes from the same population with equal mean. The p-values are given in the following tables. A p-value smaller than 0.05 indicates that the difference is significant meaning that the H0-Hypothesis is rejected with a significance level of 5%. In these cases, the values with a significant difference are highlighted in light blue in the tables.

Table E3 P-Values based on the t-test comparing covariate force direction

	supine vs prone
peak lin. displ.	$3.7 \cdot 10^{-5}$
peak lin. acc.	$2.0 \cdot 10^{-4}$
time to peak lin. acc.	$1.2 \cdot 10^{-9}$
peak rot. acc.	$6.2 \cdot 10^{-4}$
time to peak rot. acc.	0.1
EMG latency SCM	0.7
EMG latency trap.	0.4

Table E4 P-Values based on the t-test comparing covariate sex

	men vs women, supine	men vs women, prone
peak lin. displ.	0.19	0.07
peak lin. acc.	0.36	$7 \cdot 10^{-3}$
time to peak lin. acc.	0.04	0.98
peak rot. acc.	0.089	0.023
time to peak rot. acc.	0.32	0.83
EMG latency SCM	0.08	0.054
EMG latency trap.	0.099	0.038

Table E5 P-Values based on the t-test comparing covariate age

	22-51 y. vs 63-71 y., supine	22-51 y. vs 63-71 y., prone
peak lin. displ.	0.36	0.28
peak lin. acc.	0.1	0.21
time to peak lin. acc.	0.048	0.51
peak rot. acc.	$4 \cdot 10^{-3}$	0.23
time to peak rot. acc.	0.43	0.63
EMG latency SCM	$6.7 \cdot 10^{-4}$	$3.5 \cdot 10^{-4}$
EMG latency trap.	0.19	0.15

References

Bayer A, Schmitt S, Günther M, et al (2017) The influence of biophysical muscle properties on simulating fast human arm movements. Computer methods in biomechanics and biomedical engineering 20(8):803–821. https://doi.org/10.1080/10255842.2017.1293663

Devane K, Johnson D, Gayzik FS (2019) Validation of a simplified human body model in relaxed and braced conditions in low-speed frontal sled tests. Traffic Injury Prevention pp 1–6. https://doi.org/10.1080/15389588.2019.1655733

Kato D, Nakahira Y, Iwamoto M (2017) A study of muscle control with two feedback controls for posture and reaction force for more accurate prediction of occupant kinematics in low-speed frontal impacts. In: Proceedings of the 25th International technical conference on the enhanced safety of vehicles (ESV), Detroit, USA

Kato D, Nakahira Y, Atsumi N, et al (2018) Development of human-body model thums version 6 containing muscle controllers and application to injury analysis in frontal collision after brake deceleration. In: Proceedings of the International IRCOBI Conference. IRCOBI Council, Athens, Greece, URL http://www.ircobi.org/wordpress/downloads/irc18/pdf-files/32.pdf

Kleinbach C, Martynenko O, Promies J, et al (2017) Implementation and validation of the extended hill-type muscle model with robust routing capabilities in ls-dyna for active human body models. Biomedical engineering online 16(1):109

Kleinbach CG (2019) Simulation of occupant kinematics using active human body models. Dissertation, Universität Stuttgart, Düren

Larsson E, Iraeus J, Fice J, et al (2019) Active human body model predictions compared to volunteer response in experiments with braking, lane change, and combined manoeuvres. In: Proceedings of the International IRCOBI Conference. IRCOBI Council, Florence, Italy, pp 349–369, URL http://www.ircobi.org/wordpress/downloads/irc19/pdf-files/50.pdf

LSTC (2016a) LS-DYNA R9.0 Keyword User's Manual Volume I. 08/29/16 (r:7883) URL http://www.lstc.com/download/manuals

LSTC (2016b) LS-DYNA R9.0 Keyword User's Manual Volume II Material Models. 08/31/16 (r:7893) URL http://www.lstc.com/download/manuals

Martynenko O, Kempter F, Kleinbach C, et al (2018) Integrated physiologically motivated controller for the open-source extended Hill-type muscle

model in LS-DYNA. In: Proceedings of the International IRCOBI Conference. IRCOBI Council, Athens, Greece, pp 239–241, URL http://www.ircobi.org/wordpress/downloads/irc18/pdf-files/35.pdf

Martynenko OV, Neininger FT, Schmitt S (2019) Development of a hybrid muscle controller for an active finite element human body model in LS-DYNA capable of occupant kinematics prediction in frontal and lateral maneuvers. In: Proceedings of the 26th International Technical Conference on the Enhanced Safety of Vehicles (ESV), Eindhoven, Netherlands, pp 1–12, URL https://www-esv.nhtsa.dot.gov/Proceedings/26/26ESV-000215.pdf

Nölle L, Lerge P, Martynenko O, et al (2022) EHTM Code and Manual. DaRUS https://doi.org/10.18419/darus-1144, URL https://doi.org/10.18419/darus-1144

Öztürk A, Mayer C, Kumar H, et al (2019) A step towards integrated safety simulation through pre-crash to in-crash data transfer. In: Proceedings of the 26th International Technical Conference on the Enhanced Safety of Vehicles (ESV), Eindhoven, Netherlands, pp 1–10, URL http://www-esv.nhtsa.dot.gov/Proceedings/26/26ESV-000257.pdf

Sugiyama T, Weber J, Sandoz B, et al (2018) Validation of a reactive finite element human body model under moderate lateral loading. In: Proceedings of the 7th International Symposium: Human Modeling and Simulation in Automotive Engineering, Berlin, Germany

Yigit E (2018) Reaktives FE-Menschmodell im Insassenschutz. Springer Fachmedien Wiesbaden, https://doi.org/10.1007/978-3-658-21226-1
